# Two promising approaches in the treatment of myocardial infarction: stem cells and gene therapy

**DOI:** 10.3389/fcvm.2025.1540066

**Published:** 2025-02-19

**Authors:** Shan Gao, Dan Li, Bingkai Wang, Hao Zhang, Lu Chen

**Affiliations:** ^1^ Medical Experimental Center, First Teaching Hospital of Tianjin University of Traditional Chinese Medicine, National Clinical Research Center for Chinese Medicine Acupuncture and Moxibustion, Tianjin, China; ^2^ Key Laboratory of Translational Research of TCM Prescription and Syndrome, Tianjin, China; ^3^ College of Traditional Chinese Medicine, Beijing University of Traditional Chinese Medicine, Beijing, China; ^4^ School of Medical Technology, Tianjin University of Traditional Chinese Medicine, Tianjin, China

**Keywords:** myocardial infarction, stem cells, cell transplantation, gene technology, therapy, safety

## Abstract

Cardiovascular diseases (CVDs), characterized by a high incidence rate and high mortality, have become the leading cause of death globally. CVDs include coronary heart disease, stroke, hypertension, and peripheral vascular diseases. In China, the death rate of CVDs ranks the first in all major diseases. At present, the main methods to treat ischemic heart disease are drug therapy, intervention and operation. These methods only alleviate symptoms of heart failure and myocardial ischemia and improve patients' quality of life by partially restoring myocardial reperfusion. Due to the extensive irreversible necrosis of myocardial cells caused by ischemia and hypoxia, these methods cannot reverse the damage, resulting in suboptimal long-term outcomes. Although mature cardiomyocytes have been proved not to be terminally differentiated cells, they have very limited ability of regeneration and proliferation, so they can not completely replace the damaged myocardium and restore the contractile function. Although heart transplantation can replace the damaged heart, its clinical application and promotion are limited by the source of donor, expensive cost, immune rejection, and ethical problems. It has become an urgent task for clinical medicine to seek new and better treatment. The main content of this paper is to explore the application of stem cells and gene technology in the treatment of myocardial infarction (MI).

## Graphical Abstract

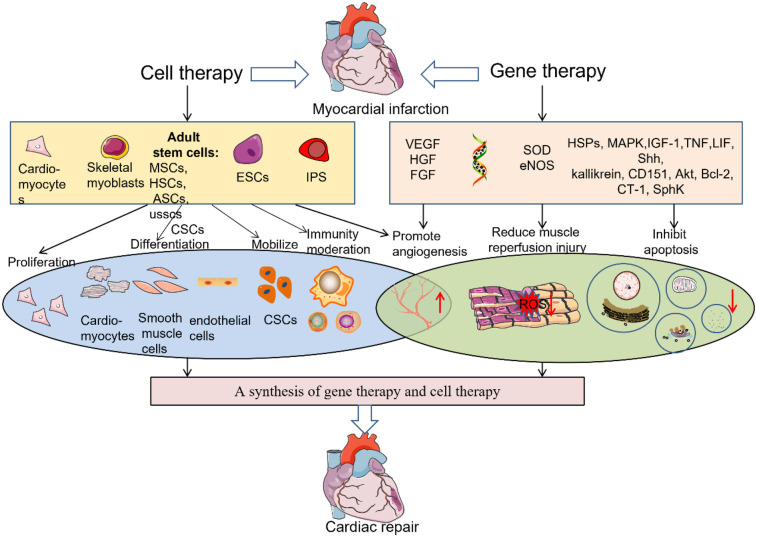

## 1 Introduction

Myocardial infarction (MI), commonly known as a heart attack, remains a leading cause of morbidity and mortality worldwide. Despite advances in emergency medical interventions such as reperfusion therapy, many patients still suffer from significant long-term cardiac dysfunction due to irreversible myocardial damage. The limited regenerative capacity of the adult heart presents a major challenge in the treatment and recovery process (1). Therefore, exploring innovative therapeutic strategies to promote myocardial repair and improve cardiac function has become a critical area of research. In recent years, two promising approaches have emerged as potential game-changers in the treatment of myocardial infarction: stem cell therapy and gene therapy (2, 3). Stem cell therapy involves the use of various types of stem cells, such as mesenchymal stem cells (MSCs) and induced pluripotent stem cells (iPSCs), to replace damaged myocardial cells and stimulate the regeneration of cardiac tissue (4, 5). These cells can differentiate into cardiomyocytes, endothelial cells, and smooth muscle cells, thereby contributing to the structural and functional restoration of the heart. Additionally, stem cells can secrete paracrine factors that promote angiogenesis, reduce inflammation, and inhibit apoptosis, creating a more favorable environment for cardiac repair (6). On the other hand, gene therapy focuses on the delivery of specific genes to modify the expression of target proteins within cardiac cells. This approach aims to enhance the intrinsic regenerative capacity of the heart by upregulating genes involved in cardiomyocyte proliferation, survival, and differentiation. Overexpression of certain growth factors or transcription factors can stimulate the endogenous cardiac progenitor cells to proliferate and differentiate into functional cardiomyocytes (7, 8). Gene therapy can also target pathways involved in fibrosis and remodeling, thereby preventing adverse structural changes in the heart following an infarction (9, 10). However, several challenges remain to be addressed, including optimizing cell delivery methods, ensuring long-term gene expression, and minimizing potential side effects (11–14). This review aims to provide an in-depth analysis of the current progress, mechanisms of action, and future prospects of these two therapeutic approaches, highlighting their potential to transform the clinical management of myocardial infarction and improve patient outcomes.

## 2 Cell therapy of myocardial infarction

Cell therapy is becoming a new strategy to avoid the adverse effects of heart disease. Many experimental and clinical studies have shown that the transplantation of cells into damaged myocardium has obtained gratifying results. Of note, these data show that the transplanted stem cells can differentiate into the main heart cell types and improve heart function. However, some clinical trials show contradictory results.

**Figure 1:**
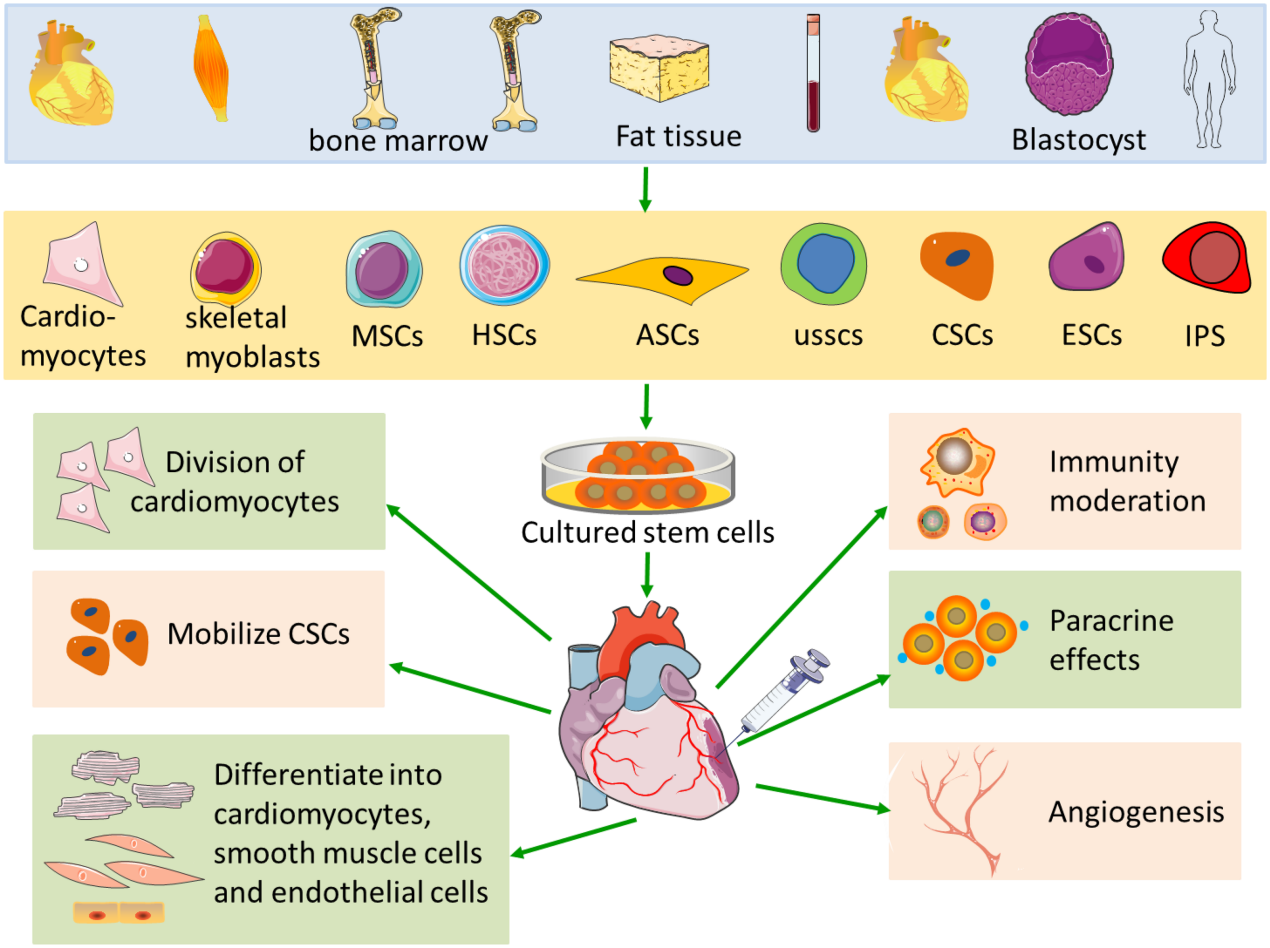
Schematic of stem cell therapy in MI. MI necessitates the use of various cell types for myocardial regeneration. Each cell type has its own unique advantages and disadvantages when applied in the context of MI. MSCs, mesenchymal stem cells; HSCs, hematopoietic stem cells; ASCs, adults stem cells; USSCs, unrestricted somatic stem cell; CSCs, cardiac stem cells; ESCs, embryonic stem cells; iPS, inducible pluripotent stem cells.

The occurrence and development of heart disease include various pathological changes of heart structure and function. MI and coronary artery disease are the most common heart diseases in Western countries. MI can lead to myocardial remodeling, including left ventricular dilation, cardiac hypertrophy, formation of fibrotic scar tissue, and reduction of myocardial cells (15). Interventional therapies can improve the patients' quality of life, but these treatments cannot prevent further heart failure (16). In animal models, cell-based therapies can not only replace dead cardiomyocytes but also inhibit cardiac remodeling. In particular, neonatal, fetal, and adult cardiomyocytes (17), skeletal myoblasts (18), human cord blood cells (19), endothelial progenitor cells (20), embryonic stem cells (21), bone marrow stem cells (22), mesenchymal stem cells (23) and induced pluripotent stem cells are used for myocardial regeneration after MI (Figure 1).

Many literatures have proved that cell transplantation is a good strategy to repair damaged myocardium. In this section, we introduce the advantages and disadvantages of different cell types used in MI (Table 1), some laboratory and clinical cell transplantation attempts, and further explore the limitations of various types of cells used for regeneration of damaged myocardium.

**Table 1:** The advantages and disadvantages of different cells types used in MI.

Cell type	Advantages	Disadvantages
Cardiomyocytes	- Fetal and neonatal cardiomyocytes - Can repair the damaged myocardium - Low risk of tumorigenicity	- The division of cardiomyocytes is not enough - Requires heart biopsy - Not used in clinical practice
Skeletal myoblasts	- Easy to obtain - High proliferative potential - Autologous transplantation - Resistance to ischemia - Not subject to ethical challenges - Low risk of tumorigenicity	- Lack of functional cardiomyocytes differentiation - Risk of ventricular arrhythmias - Low long-term survival rate - Invasive isolation procedure
Mesenchymal stem cells (mscs)	- Easy to isolate and expand *in vitro* - A wide range of differentiation potential - Low immunogenicity -immunomodulatory properties	- Low engraftment after transplantation
Hematopoietic stem cells (HSCs)	- The potential to differentiate into cardiomyocytes	- Contradictory results
Adipose-derived stem cells (ASCs)	- Pluripotent, immature, and self- renewing - Not subject to ethical challenges - Can be harvested in an effective manner	- Contradictory results
Cord blood stem cells (CBSCs)	- Contains a variety of stem cells - Easy to collect and preserve	- Immune rejection - Require storing cells at birth - Untested in clinical practice
Cardiac stem cells (CSCs)	- Autologous transplantation - Pluripotent - Feasibility in clinical trials - Low risk of tumorigenicity	- Renewal rate is low - Access from invasive myocardial -biopsies
Embryonic stem cells (ESCs)	- Pluripotent and undifferentiated cells - Self-renewal for a long time *in vitro* - Can differentiate into specific cells	- Ethical issues - Teratoma formation - Require the use of immunosuppressive drugs - Lack of availability
Inducible pluripotent stem cells (iPSCs)	- Totipotency and self-renewal - Can differentiate into three germ cells - Availability of source tissue - Avoiding the ethical issues - Reducing the use of immunosuppressive drugs.	- Teratoma formation - Limited genome editing technology - Untested in clinical practice

### 2.1 Cardiomyocytes

During the development and formation of the heart in the embryonic stage, cardiomyocytes rapidly proliferate and differentiate into form mature cardiomyocytes, which constitute the structure of the heart (24). In most mammals, during the last round of replication, cardiac myocytes undergo DNA synthesis and mitosis without cytoplasmic division, while cells in other organs undergo cytoplasmic division at the same time to form two different daughter cells (25). In adult cardiomyocytes, there are often mononuclear, binucleate, or even trinuclear cardiomyocytes with undifferentiated cytoplasm. Previous research has long suggested that in response to adult heart injury, cardiomyocytes primarily rely on hypertrophy rather than cell division to meet the increased demand for blood supply. This is characterized by an enlargement of cardiomyocyte volume. However, recent studies have challenged this traditional view by demonstrating that cardiomyocytes can indeed undergo division, not only in normal myocardium but also in infarcted regions. This new evidence suggests a more complex and dynamic role of cardiomyocytes in the context of cardiac injury and repair (26, 27). Neonatal human hearts might have the capacity of repairing myocardial injury and recovering cardiac function after MI (28). However, the division of cardiomyocytes after myocardial injury is not enough to repair the damaged myocardium. So far, numerous studies have shown that in many heart diseases, including acute MI, ischemic heart failure, chronic myocardial ischemia and hereditary heart disease, transplantation of neonatal, fetal, or adult cardiomyocytes is effective, and these cells can repair the damaged myocardium after transplantation (29). These results show that the transplanted cells can successfully differentiate into the myocardium and improve contractile function. Fetal, neonatal, and adult cardiomyocyte transplantation is likely to successfully repair the damaged heart. Compared with adult cardiomyocytes, fetal and neonatal cardiomyocytes have a better effect on repairing damaged myocardium. This is because the genetic material of neonatal and fetal cardiomyocytes is not mature yet, and they have stronger plasticity compared with adult cardiomyocytes. However, these kinds of cardiomyocytes cannot be used in clinical practice, because the acquisition of these cells requires heart biopsy.

### 2.2 Skeletal myoblasts

Skeletal muscle is one of the three main muscle types in the human body. Skeletal muscle fibers are the basic functional elements of these cells; they produce the mechanical forces necessary for muscle contraction, similar to cardiomyocytes. However, unlike cardiomyocytes, skeletal myoblasts still maintain their regeneration ability after injury. Given this ability, skeletal myoblasts seem to be a suitable new source of cardiac cell therapy. Besides, autogenous skeletal myoblasts were used to solve the problem of the source of donor cells. The source of donor cells has been limiting the application of adult cardiomyocytes and neonatal cardiomyocytes. To sum up, skeletal myoblasts have the advantage of resistance to ischemia, autologous transplantation, and high proliferative potential (30).

Previous studies have shown that transplantation of autologous skeletal myoblasts into the damaged heart can improve the systolic and diastolic properties of the damaged heart. Some clinical studies found skeletal myoblasts transplantation to be a feasible and sufficient treatment for MI, playing an important role in improving cardiac function by suppressing fibrosis, reducing left ventricular wall stress, and improving regional wall motion (31, 32). However, in another study of autogenous transplantation of skeletal myoblasts, the phase I clinical trial failed to prove that transplantation of skeletal myoblasts can improve the condition of patients with ischemic cardiomyopathy. This experiment found that compared with the placebo group, the frequency of arrhythmias in the cell transplantation group increased (33). These side effects observed in patients limit the clinical application of skeletal myoblasts.

### 2.3 Adult stem cells

#### 2.3.1 Mesenchymal stem cells (MSCs)

Adult stem cells exist in all organs of the body. The best source of adult stem cells is bone marrow (BM). Bone marrow stem cell groups commonly used in experiments and clinical include hematopoietic stem cells (HSCs), c-kit^+^ cells, SCA^+^ cells, MSCs, and bone marrow-derived mononuclear cells (BMMNC) (34). MSCs derived from bone marrow are easy to differentiate and proliferate *in vitro*. *In vitro* and vivo experiments show that they can differentiate into osteoblasts, chondrocytes, monocytes, and adipocytes. The repair mechanisms of MSCs in MI might include autocrine effects, paracrine effects, and endocrine-like effects. The autocrine effects of MSCs could promote survival, proliferation, and possible differentiation. The paracrine pathway could secrete trophic factors acting on adjacent cells, and promote angiogenesis and inhibit fibrosis. Endocrine-like pathways secreted trophic factors circulating in vascular or lymphatic systems to induce the repair effect.

In fact, many *in vitro* studies have shown that MSCs have the potential to differentiate into cardiomyocytes in the presence of retinoic acid, DMSO, and 5-aza, or when co-cultured with cardiomyocytes. Animal experiments showed that MSCs transplantation after MI inhibited ventricular remodeling and improved cardiac function. The remarkable effect of MSCs is mainly due to its paracrine effect on host cardiomyocytes, rather than cell fusion and differentiation. Moreover, MSCs overexpressing survival-promoting factor Akt can protect myocardium 72 h after transplantation. Recent studies showed that the secretion of exosomes played an important role in the effects of cardioprotection. Exosomes transferred bioactive molecules including miRNAs and proteins between cells, which helped improve angiogenesis, proliferation, and suppress apoptosis (35). An analysis of mesenchymal stem cell proteomes successfully identified the MSC-specific nascent proteins in the infarcted hearts, which might help to reflect the reparative effects of MSCs (36).

#### 2.3.2 Hematopoietic stem cells (HSCs)

Another group of bone marrow-derived stem cell is HSCs, which have the potential to differentiate into multi-lineal blood cells including lymphocytes and stromal cells. There was a study proved that HSCs possessed the unique ability to differentiate into cardiomyocytes (37). However, more evidence showed HSCs could only differentiate into hematopoietic lineage cells but not into cardiomyocytes (38, 39). The specific proteins of HSCs are CD34^+^ and CD133^+^ in human, Lin^+^, and c-kit^+^ in mice. At different stages of differentiation, HSCs no longer express these cell surface markers (40). HSCs are used for myocardial repairation in different animal models. However, these data have produced contradictory results. In one study, mice were ligated with the left anterior descending branch and then transplanted with HSC derived bone marrow cells with both Lin and c-kit positive. After 9 days of cell transplantation, endothelial cells (ECs) and smooth muscle cells (SMCs) from donors could be detected. Moreover, compared with the control group, the ventricular function of the treatment group was significantly improved. However, this finding has been questioned by subsequent articles, which show that after hematopoietic stem cell transplantation, no myocardial regeneration or improvement of cardiac function has been found (41). In a clinical study of chronic MI patients, intramyocardial injection of specific bone marrow HSCs in the injury area enhanced myocardium regeneration and improved cardiac contractility, and without adverse side effects events occurred (42). In general, HSCs are not considered as an ideal stem cell group for myocardial regeneration.

#### 2.3.3 Adipose-derived stem cells (ASCs)

It has been proved that stem cells in adipose tissue are pluripotent, immature, and self-renewing. ASCs are not subject to ethical challenges and can be harvested in an effective manner such as surgical scraps from the surgical specimens. ASCs can be isolated from human and animal adipose tissues including abdomen, thigh, and upper limb, and play roles in tissue regeneration and repair by secreting growth factors, cytokines, neurotrophic factors, and angiogenic factors (43). Many studies have shown the advantage of injecting ASCs into damaged myocardium. For example, in a study, ASCs injected into the myocardium can differentiate into cardiomyocytes, and preconditioned ASCs attenuate cardiac fibrosis after MI (44). In LPS-induced cardiomyocyte damage, ASCs showed a cardioprotective effect (45). Other studies have shown that transplanted ASCs play a role through the secretion of growth factors, which can mobilize the stem cells of the heart itself to reach the damaged site and repair the damaged myocardium (46).

#### 2.3.4 Cord blood stem cells (CBSCs)

CBSCs are very promising for tissue repair and regeneration because cord blood (CB) contains a variety of stem cells, and CB is easy to collect and preserve. In addition to HSCs and MSCs, CBSCs contain many adult stem cells that are not limited by application. Although the number of these cells is small, they can be easily separated and expanded without losing the pluripotency of cells. *In vivo* and *vitro*, CBSCs can differentiate into osteoblasts, chondrocytes, adipocytes, and nerve cells (47). The advantages of this cell as a cardiac therapeutic cell have been proved in the porcine model of MI. This experiment shows that the transplanted CBSCs has the ability of myocardial regeneration (48). In the model of MI of rats, CBSCs can repair damaged myocardium and induce angiogenesis, to improve heart function (19, 49, 50). Although these cells have the potential of myocardial regeneration, they have not been used in the clinical treatment of heart disease. Moreover, to avoid immune rejection, the use of CBSCs in clinical trials requires patients store cells at birth for later transplantation.

#### 2.3.5 Cardiac stem cells (CSCs)

Studies have shown that the heart itself contains some natural multifunctional cardiac stem cells (CSCs). CSCs have been successfully isolated from mouse and human heart biopsy tissues and cultured *in vitro*. After transplanted into the infarcted heart, these cells can differentiate into cardiomyocytes, vascular smooth muscle cells (VSMCs), and ECs (51). However, the renewal rate of CSCs is low and decreases from 1% at age 25 years to 0.45% at age 75 years (27). A clinical trial involving patients with MI demonstrated the safety, feasibility, and potential therapeutic effect of adult cardiac stem cell transplantation (52). In animal models, the results have been published to prove the effect of CSCs on cardiac repair and regeneration (53). Nevertheless, other studies showed that CSCs didn't harbor the ability for cardiomyocyte regeneration or improve cardiac function (54, 55). As CSCs proliferate at a low frequency, methods that motivate cardiomyocyte proliferation may become a crucial research field in the future.

### 2.4 Embryonic stem cells (ESCs)

ESCs are pluripotent and undifferentiated cells, which can maintain the ability of self-renewal for a long time *in vitro*. In addition, under specific physiological or experimental conditions, ESCs can differentiate into specific cells, including nerve cells, ECs, VSMCs, and cardiomyocytes (56). The primitive three-dimensional cell structure formed by ESCs *in vitro* is called embryoid body (EBS). Embryoids contain cells from all three germ layers, such as cardiomyocytes, neural cells, and hematopoietic cells (57). Current studies show that ESCs can be induced to produce paracrine and autocrine effects, release cytokines, thus affecting the endogenous cell microenvironment and improving heart function. The exact factors and mechanisms are not clear, and some promising intermediates are being further studied. For example, tissue inhibitor of metalloproteinase1 (TIMP1), a growth factor of fibrosis and anti-apoptosis, has been shown to promote myocardial repair. Although the research of TIMP-1 has achieved gratifying results (58), its mechanism is still under study.

There are two ways for ESCs to help cardiac regeneration after heart injury: one is to form the main types of cardiac cells through differentiation, the other is to release anti-apoptosis and anti-fibrosis factors. However, the application of ESCs in heart cell therapy is restricted by ethics, because these stem cells were isolated from embryos. In addition, the histocompatibility and teratoma formation should be considered in the application of embryonic stem cells. The use of ESCs requires the use of immunosuppressive drugs to overcome the differences in histocompatibility and inhibit cell rejection after transplantation. Teratomas are stem cell-derived tumors, which are a mixture of cells from three germ layers. They are differentiated cell clusters formed by undifferentiated or out of control differentiation of stem cells. There is a potential risk of forming teratomas after ESCs transplantation (59, 60). The good news is that current studies have shown teratoma formation *in vivo* can be avoided when the number of ESCs transplanted is limited to 5 × 10^4^ or less (61).

In a pig model of MI, human ESCs-derived cardiomyocytes could regenerate the infarcted cardiomyocytes, but give rise to ventricular tachyarrhythmias at the same time (62). To solve the problem of the low rate of stem cell survival that injected into the myocardium, a study of co-transplanting biomaterial with ESC-derived cardiomyocytes into the infarcted myocardium of rat MI model was performed (63). And the results showed that the combination therapy improved cardiac function and lowered the rates of induced arrhythmias.

### 2.5 Inducible pluripotent stem cells (iPSCs)

Yamanaka and his team (64) induced cell groups with similar characteristics to ESCs, induced pluripotent stem cells (iPSCs). Through retroviral transduction, they made adult mouse fibroblasts express four transcription factors: (1) krueppel like factor 4 (KLF4), (2) SRY related HMG box transcription factor (Sox2), (3) octamer binding transcription factor 3/4 (OCT3/4), and (4) myeloma gene (c-myc). These embryonic-like cells show the characteristics of embryonic stem cells, such as the expression of four cell surface markers, the formation of embryonic stem cells like clones, totipotency, and the ability to self-renew. Moreover, iPSCs can differentiate into cells of the three germ cells, including hematopoietic, neural stem cells and cardiomyocytes (65). And compared to ESCs, autologous iPSCs transplantation has the advantage of avoiding the ethical issues and immunological rejection.

iPSCs derived from fibroblasts injected into the heart confirmed the potential of these cells for cardiac cell therapy, which improved the contractile function of the ischemic heart. These iPSCs spontaneously integrate into the host myocardium and differentiate into major cardiomyocyte types. Besides, iPSCs cells have been obtained by expressing OCT3/4, Sox2, KLF4, and c-myc in H9C2 cells (66). Some studies have reported the benefit of iPSCs-derived cardiomyocytes in transplantation for heart regeneration in mouse, rat, or pig MI models (66–68). When transplanted into the infarcted heart, this kind of cells can reduce the loss of myocardial cells, reduce the formation of fibrosis scars, and significantly improve the heart function. In a rat MI model, co-transplanted adipose tissue derived- micro vessels with human induced pluripotent stem cells-derived cardiomyocytes could improve the survival of iPSCs-derived cardiomyocytes and enhance the functional recovery of the heart. Suggesting a method help to increase the viability of stem cells, promote cardiomyocyte maturation and decrease arrhythmogenicity (69). In addition, these cells can provide specific iPSCs for patients, which are very useful in the establishment of disease models and drug screening for various diseases (70), so they bring great hope for regenerative medicine.

However, before any clinical application, the safety of iPSCs must be fully considered. C-myc is a well-known protooncogene, which can activate the expression of tumorforming genes in host tissues (71). The current research can get iPSCs without using the c-myc gene, but the reprogramming efficiency of this iPSCs is quite low (72). In addition, to avoid the risk of insertion mutagenesis, non-viral iPSCs are being studied (73–75). In these studies, iPSCs were induced from adult cells by small molecules or recombinant proteins, such as polyarginine. Other studies have also involved the use of non-viral vectors or non-integrated attachment vectors that are not integrated into the genome. These methods can induce pluripotency of cells without using genetic tools.

## 3 Gene therapy for MI

In 1989, transgenic technology was first introduced into the cardiovascular field. Although many basic and clinical studies have been carried out, the clinical application of transgenic technology needs further verification. This is because, in most clinical studies, the inherent problem of immunogenicity of recombinant virus vectors is difficult to avoid. Gene therapy provides a very attractive solution for the treatment of ischemic heart disease. It can continuously provide therapeutic protein for the focus and may reverse the pathophysiology of AMI. The use of new gene structures for modification enables gene expression to be turned on or off according to the intracellular environment, minimizing waste caused by unrestricted protein synthesis or inhibition. Based on stem cell therapy, combined with gene therapy, can promote myocardial regeneration, and has a higher success rate. Therefore, gene therapy may delay or even avoid the need for heart transplantation by reversing the pathology. This section will discuss the research progress of gene therapy for MI.

### 3.1 Concept of gene therapy

Gene therapy is to compensate or correct diseases caused by gene defects and abnormalities by introducing foreign normal genes into target cells, so that the products of foreign gene expression can treat the disease.

According to the function of inserting genes, gene therapy can be divided into three categories: (1) Substituting or repairing for missing or defective genes, such as, AAV-CRISPR/Cas9-mediated gene correction can partially mediate LDLR expression and effectively ameliorate AS phenotypes in mutants, providing a potential therapeutic approach for the treatment of patients with familial hypercholesterolemia (76); (2) Enhancing endogenous physiological function by promoting gene expression, such as improving atherosclerotic vascular endothelial cells by overexpression of eNOS gene; (3) Destroy the expression or function of harmful endogenous genes, such as using antisense oligonucleotides (ODNs) to destroy E2F transcription factors to inhibit AS.

#### 3.1.1 Non-viral vector

The anionic liposome is a kind of common non-viral vector, which transfers genes by entering host cells through encapsulation. Liposome is almost non-toxic and non-immunogenic, so it is a relatively good carrier to transfer DNA into cells. However, the DNA carried by the liposome will be transported to the lysosome after being swallowed into cells, which will lead to low transfection efficiency. In response to this limitation, scientists have developed a liposome vector for fusion genes (77).

#### 3.1.2 Virus vector

Viral vector is a kind of replication-defective viral plasmid, which only retains the ability to enter the target cell and transfer its genetic material into the chromosome of the target cell. According to their sources, virus vectors can be divided into the following categories:

##### 3.1.2.1 Retroviral vector

Retrovirus is a kind of RNA virus, which can integrate the transferred gene into the host chromosome. However, gag, pol, and env genes are integrated into packaging cell lines such as 293 T cells to synthesize capsid proteins and polymerases necessary for virus replication. Retroviral vectors have many advantages, including a long time of transgene expression, large capacity of DNA coating, high virus titer, and so on. However, its ability to infect non-dividing cells is weak, which limits its application to a certain extent.

##### 3.1.2.2 Adenovirus vector

The gene of adenovirus vector is a double-stranded DNA molecule with a length of 36 KB. Adenovirus vector is the main vector for gene therapy of cardiovascular diseases *in vivo*. Adenovirus vector has many advantages: (1) It is easy to prepare; (2) It has a high virus titer; (3) It has a strong gene transfer effect; (4) It has a large capacity of covering DNA; (5) It is superior to retrovirus in that it can infect both static and slow dividing cells. However, the adenovirus vector itself has some shortcomings. The transgenic expression time of adenovirus as a gene vector is short, and can trigger the immune response of the host.

##### 3.1.2.3 Adeno-associated virus vector

Adeno-associated virus is a small DNA virus, it must rely on adenovirus or other viruses to replicate. Adeno-associated virus can carry 3–4 kb foreign gene. Similar to adenoviruses, adeno-associated viruses can infect both stationary and nondividing cells.

Gene therapy can synthesize or inhibit a specific protein, leading to changes in the structure or function of the target tissue. To carry out gene therapy, the first step is to design a stable sequence of genes of interest with promoter/enhancer. Then, the vector containing the sequence was constructed. Then, the target gene can be expressed by transferring the gene into the target cell.

The efficiency of naked plasmid DNA transfection is very low, but the target cells can be transduced by adeno-associated virus, retrovirus, and non-viral vector system. The special medical catheter can inject the carrier with the target gene into the coronary artery or the heart muscle. Besides, pericardial administration and intramyocardial injection through minimally invasive surgery have been used.

### 3.2 Gene therapy for promoting angiogenesis

#### 3.2.1 Gene therapy of vascular endothelial growth factor (VEGF)

VEGF may be the most concerned growth factor, which can promote the angiogenesis of the ischemic heart. VEGF binding to specific receptors on the surface of endothelial cells plays an essential role in the process of angiogenesis. The mammalian genome encodes five subtypes of the VEGF family, which are VEGF-A, VEGF-B, VEGF-C, VEGF-D, and placental growth factor (78). VEGF-A and VEGF-B conduct signals through VEFG receptor 1 and VEFG receptor 2, regulating vascular physiological function and development (79).

VEGF-A plays a key role in cardiac angiogenesis. The transcripts encoding VEGF-121 and VEGF-165 can be detected in most tissues and cells expressing the VEGF gene. Gene therapy for VEGF-165 is considered highly effective in promoting angiogenesis (80). VEGF-165 gene can induce obvious angiogenesis and improve the short axis contraction rate after MI in rats or rabbits by non-viral therapy (81). In the pig or dog models of MI, VEGF-165 can decrease the infarct area, improve the left ventricular contractility (82), increase cardiac wall thickness and strength (83), and improve myocardial survival rate (84), thus significantly improving the overall function of the heart.

In a clinical study, VEGF gene therapy didn't improve myocardial abnormalities but show anti-ischemic effects (85). In another study, there was a significant improvement in myocardial blood flow and angina pectoris symptoms (86). VEGF gene therapy could also mobilize the immune system and endothelial progenitor cell (87) and seemed the long-term safety (11).

Gene therapy to promote angiogenesis is not without defects, and the unregulated expression limits the effectiveness and safety of VEGF gene therapy. To solve this problem, the gene structure of expression switch regulated by extracellular environment is under study.

#### 3.2.2 Gene therapy of hepatocyte growth factor (HGF)

HGF is secreted by mesenchymal cells, which can play various roles in epithelial target cells. HGF binds to the tyrosine kinase receptor on the surface of endothelial cells, which affects the migration, proliferation, protease expression, infiltration, and angiogenesis of endothelial cells.

In animal models of heart diseases, HGF gene therapy showed the abilities of decreasing the inflammatory levels, improving cardiac function (88), increasing arteriole density, and angiogenesis (89). HGF could also inhibit apoptosis (90) and improve the contractile ability of the heart (91). Combined application of fibroblast growth factor-2 and HGF showed a more potent and durable effect of angiogenesis, and effectively reduced cardiac remodeling and improved left ventricular functions (92). HGF gene therapy combined with ultrasound-mediated microbubble breaking technology can improve the rat heart function and the efficiency of gene transfection in MI model (93). HGF-expressing MSCs improved angiogenesis, cell viability, and cardiac function (94, 95), indicating a strategy that could be used to enhance the efficacy of cell therapy. In clinical trials of patients showed that adenovirus carrying HGF is safe and potentially effective in enhancing echocardiographic indexes (96, 97), but the sample size was too small. Therefore, the results need to be confirmed in studies with a larger sample size, and a great deal of work is necessary for the clinical practice of HGF gene therapy in MI.

#### 3.2.3 Gene therapy of fibroblast growth factor (FGF)

Fibroblast growth factors (FGFs) bind to tyrosine kinase receptors, regulate cell mitosis and cell survival. FGFs promote tissue growth and affect cell proliferation and division (98). FGF family members are classified according to their affinity ligands and tissue distribution. FGF-1 and FGF-2 promote endothelial cell proliferation and tube formation. FGF-4 and FGF-5 are secretory FGFs, while FGF-1 and FGF-2 are mainly intracellular, so FGF-4 and FGF-5 have greater advantages, they have paracrine and endocrine functions.

In a mice model of MI/R, FGF-1 played roles in cardio-protection through protecting cardiac functional recovery and increasing cell survival of ischemia heart (99). In MI mouse models, FGF induced the accumulation of HIF-1*α*, promotion of angiogenesis and inhibition of apoptotic effects in cardiac endothelial cells, and improved cardiac remodeling (100). FGF could also reduce oxidative stress (101), attenuate cardiac fibrosis (102), and FGF21 showed antiarrhythmic property (103). In the pig model of MI, FGF-4 gene therapy can enhance perfusion and improve the dysfunction caused by MI (104). FGF-5 gene therapy can not only improve blood supply (105) but also improve local myocardial dysfunction by stimulating mitosis of myocardial cells (106).

### 3.3 Gene therapy to reduce muscle reperfusion injury

#### 3.3.1 Gene therapy and oxidative stress

Among many cardiovascular diseases, including MI, myocardial ischemia/reperfusion injury, atherosclerosis, endothelial dysfunction, restenosis, hypertension, cardiomyopathy, and heart failure, oxidative damage is the most serious injury. Reactive oxygen species (ROS) produced in R/I injury not only cause lipid peroxidation and protein oxidation but also affect calcium channels induced by various Calmodulins, such as ryanodine receptors (RyR), sarcoplasmic reticulum (SR) Ca2^+^ ATPase (SERCA), and 1,4,5-inositol triphosphate (IP3), thus increasing calcium entry into cardiomyocytes and causing cell damage (107). The expression of many antioxidant enzymes, including superoxide dismutase (SOD), glutathione peroxidase (GPX), catalase, or heme oxygenase-1 (HO-1), is induced by inflammation or other stimuli. The production of these antioxidant enzymes is a protective mechanism to eliminate ROS. SOD has three subtypes to catalyze the disproportionation of superoxide anion, thus reducing the oxidative damage of cells (108). Gene therapy with extracellular isoform of SOD attenuated myocardial injury through increasing antioxidant enzymes in rabbit and mouse model of MI (109, 110).

#### 3.3.2 Gene therapy of endothelial nitric oxide synthetase (eNOS)

In the CVDs, nitric oxide (NO) is a signal molecule with multiple protective functions (111). In mammals, it is synthesized by L-arginine under the action of endothelial nitric oxide synthetase, and NO mainly plays a protective role on the myocardium. Adenovirus-mediated human eNOS gene therapy after MI operation could reduce MI area, apoptosis, JNK phosphorylation level, caspase-3 activity, and improve cardiac remodeling (112). ENOS can reduce the level of fibrosis, improve endothelial progenitor cells and vascular function after MI (113). Gene engineering with eNOs of EPCs from patients with coronary artery disease showed improvement in EPCs migration, differentiation, and angiogenesis *in vitro*, possibly through a paracrine effect (114). A combinational approach of hydrogel loaded with ADSCs and plasmid DNA-eNOs was used to treat MI rats, and the results showed that this approach could improve the heart functions, including infraction size, fibrosis area, and vessel density (115). Adenoviral transfected of human eNOS gene into MSCs remarkably enhanced capillary density and hemodynamic parameters (116).

### 3.4 Gene therapy for inhibition of cardiomyocyte apoptosis

#### 3.4.1 Heat shock proteins (HSPs)

HSPs act as molecular chaperones, responsible for protein folding, intracellular protein transport, denaturation, or modification of other proteins under heat or other stimuli. MIMI Overexpression of HSP20 improved cardiac contractile function, increased SR Ca-Cycling in cardiomyocytes, and played a cardioprotective role in heart disease (117). In a MI model, overexpressed HSP20-mediated cardiomyocyte exosomes increased cell survival by activating Akt signaling pathway and decreasing tumor necrosis factor-α (TNF-α) and interleukin-1β (IL-1β) (118). HSP27 significantly protected the heart from apoptosis or oxidative stress against MIRI (119). Overexpression of HSP60 protected myocytes from apoptosis by decreasing cytochrome c release, caspase-3 activation, and enhancing ATP recovery (120). HSP70 gene pre-therapy can reduce the area of MI in rabbits (121). Therefore, gene therapy with HSP can reduce the apoptosis after myocardial ischemia, and HSPs can protect the heart after MI.

#### 3.4.2 Mitogen-activated protein kinase (MAPK)

The amplification of mitogen-activated protein kinase (MAPK) is composed of extracellular signal-regulated protein kinase (ERK), p38 kinase, and c-Jun N-terminal protein kinase (JNK). MAPK is a key regulator of cell survival and death. Poor ventricular remodeling after infarction is associated with decreased p38 signal. p38-regulated/activated protein kinase deficiency decreased angiogenesis and enhanced myocardial fibrosis and cardiomyocytes apoptosis (122). Co-transfection of wild-type (WT) p38 kinase and activated MKK3b (the protein is the activator upstream of p38 kinase) reduced MI area and apoptosis, increased capillary density, decreased fibrosis, and improved ejection fraction in rat MI/R model (123). But p38 inhibitors were also reported to improve cardiac performance (124). These results suggested the bidirectional effects of p38 at different time points of the disease process.

#### 3.4.3 Insulin-like growth factor-1 (IGF-1)

IGF-1 is mainly secreted by the liver. It is an endocrine hormone, but it is produced in the form of paracrine/endocrine in the target tissue. IGF-1 is produced by the stimulation of growth hormone, and its secretion is inhibited when it is malnutrition and insensitive to growth hormone. IGF-1 can inhibit programmed cell death, activate AKT signaling pathway, and promote cell growth and proliferation. IGF-1 overexpressed mesenchymal stem cells can promote the migration, growth, and proliferation of bone marrow-derived mesenchymal stem cells by activating the AKT/SFRP2 signaling pathway, and inhibit cell apoptosis (125). In a porcine AMI model, microencapsulated IGF-1 therapy reduced the infarct area and improved cardiac function (126).

### 3.5 Other gene therapy to inhibit cardiomyocyte apoptosis

#### 3.5.1 Tumor necrosis factor (TNF)

TNF is produced by activated macrophages. It can activate apoptosis, inflammation and inhibit tumor formation through the TNF receptor. Soluble TNF- α receptor 1 (sTNFR1) is an antagonist of TNF. In the MI model of mice, sTNFR1 gene therapy can reduce the infarct area and improve the heart function (127). However, in a human study, TNF-α antagonism decreases systemic inflammation but enhances platelet activation, and is not recommended for therapeutic strategy in patients of AMI (128).

#### 3.5.2 Leukemia inhibitory factor (LIF)

LIF is an IL-6-related cytokine, which can regulate cell differentiation, growth, proliferation, tissue regeneration, inflammation, and immune response (129). In the ischemic border area, the transfer and overexpression of the LIF gene can reduce the degree of fibrosis, increase the left ventricular wall thickness, reduce the apoptosis in the ischemic area, and induce neovascularization (130). In addition, LIF partly mobilizes stem cell-derived cardiomyocyte regeneration through the activation of cardiac stem or precursor cells (131).

#### 3.5.3 Sonic hedgehog (Shh)

Shh is a protein-related to the Hedgehog signaling pathway in mammals, which plays a key role in organ formation and development. Injection of naked DNA encoding the human Shh gene into ischemic myocardium showed that it can protect ventricular function by enhancing angiogenesis, inhibiting apoptosis, and inhibiting fibrosis (132). In pig models of myocardial ischemia reperfusion, the activation of Shh improved cardiac function, alleviated reperfusion arrhythmias, and decreased infarct area (133).

#### 3.5.4 Kallikrein

Kallikrein kinin system is a system that can control blood pressure, cause pain and inflammation. It is mediated by vasorelaxant kinin and lysine bradykinin, which are produced by the hydrolysis of their precursor kininogen by kallikrein. In the MI model of rats, gene therapy of kallikrein can increase capillary density, reduce apoptosis, reduce endothelial dysfunction and improve cardiac function after MI (134), and the mechanism is possibly concerned with the kinin B2 receptor-Akt-GSK-3beta and VEGF signaling pathways (135).

#### 3.5.5 Cd151

The cell surface protein encoded by the CD151 gene plays an important role in cell development, growth, activation, and migration. In pig and rabbit models of MI, CD151 gene therapy can promote angiogenesis and improve heart function (136–138).

#### 3.5.6 Akt protein kinase

Akt protein kinase family members are related to the signaling pathways of cell survival and apoptosis inhibition. AKT1 is also associated with angiogenesis. Adenovirus mediated Akt gene therapy can reduce the infarct area, enhance myocardial contractility, and intracellular calcium content of heart (139). Therefore, Akt gene therapy has the potential to treat heart failure.

#### 3.5.7 Bcl-2 protein

Bcl-2 plays an antiapoptotic role in mitochondria by neutralizing Bax and inhibiting the release of cytochrome c. Overexpression of Bcl-2 can promote cell survival and inhibit apoptosis. In the ischemia-reperfusion model of mice, compared with the wild type mice, the transgenic mice with super surface human Bcl-2 gene reduced the MI area and improved the ejection fraction (140). BCL2 gene therapy can reduce apoptosis, the extent of ventricular dilation, and the reduction of wall thickness in the rabbit model of ischemia-reperfusion (141).

#### 3.5.8 Cardiac Troponin-1 (CT-1)

CT-1, a member of the interleukin-6 family, can protect the heart from ischemia-reperfusion injury and play a key role in cardiac repair and cardiac hypertrophy. In the mouse model of MI, CT-1 gene therapy inhibited apoptosis, reduced the area of MI, reduced the activity of caspase, and improved the ventricular pressure index (142). And CT-1 could increase the engraftment of mesenchymal stromal cells to improve the effect of stem cell therapy (143).

#### 3.5.9 Sphingosine kinase (SphK)

SphK is a lipid kinase, which is related to the metabolism of sphingosine. SphK1 catalyzes the formation of sphingosine-1-phosphate (S1P), which activates eNOS through its receptor S1P1, induces endothelial cell chemotaxis, and maintains vascular integrity (144). In the rat model of MI, SphK1 gene therapy can maintain the systolic and diastolic function of the heart and improve the peak systolic flow rate (145).

Therefore, gene therapy with overexpression of TNF, LIF, Shh protein, kallikrein, CD151, Akt, Bcl-2, inhibitor of apoptosis, CT-1 and SphK can improve various pathological parameters related to ischemic heart disease.

## 4 Discussion

### 4.1 Clinical trial of stem cell transplantation

Many clinical trials have used cell transplants to treat heart disease. Some clinical trials successfully evaluated the feasibility of using skeletal myoblasts to treat patients with ischemic heart failure (31, 32). In addition, the increased frequency of arrhythmia in the experimental group makes the safety of the application of skeletal myoblasts to be verified. ESCs-derived cardiac progenitors embedded into a fibrin scaffold were transplanted into the infarct heart of the patient (146). Some clinical trials use bone marrow derived stem cell groups to test the safety and feasibility of their therapeutic applications. Some clinical trials do not choose to use BMCs, but a specific type of cells in BMCs, such as CD34^+^ stem cells (147). The effect of intramyocardial injection of bone marrow-derived cells on cardiac function in patients with acute myocardial infarction (AMI) was studied (148, 149). The data showed that during the 6-month follow-up period, the systolic function of patients injected with BMCs was significantly improved. In another clinical trial, 200 patients with AMI were injected with bone marrow-derived mononuclear cells, bone marrow-derived CD34+CXCR4+ cells, and placebo. This study did not find a significant improvement in the left ventricular ejection fraction (LVEF) in the treatment group. However, the authors suggest that cyto-therapy may be more appropriate for patients with more severe conditions (150). There was a study demonstrated the safety and feasibility of intracardiac injection of circulating progenitor cells (CPCs) or bone marrow-derived progenitor cells (BMC) (151). In an experiment, 120 patients with AMI with PCI were treated by bone marrow mononuclear cells (BMCs), the results showed the BMCs had no significant effect on improvement of left ventricular (LV) function (152). In another trial, there were no definitive answers acquired regarding the effect of bone marrow-derived mononuclear cell because of the unexpected low recruitment and event rates (153). The use of allogeneic cardiac stem cells- cardiosphere-derived *cells* (CDCs) were also utilized to treat patients with heart disease and showed the safety and feasibility of these cells (52).

In general, the conflicting results observed in various adult stem cell transplantation experiments indicate that cell therapy for MI needs further study. Divergent results indicate a lack of knowledge about the mechanisms by which cells survive and function in the repair of infarcted myocardium. Despite advances in cardiac cell therapy over the past decade, we are still looking for the best cell types for cardiac repair. It is encouraging that many factors have been found to promote stem cells to differentiate into cardiomyocytes. So far, the genetic and molecular mechanisms of cell homing, myocardial differentiation, and myocardial repair after cell transplantation are still unknown. Therefore, the research in this field needs more attention. More importantly, the safety problems found in various clinical trials, including the increase of arrhythmia attack frequency, the formation of teratoma, and the immune rejection of transplanted cells, need to be solved through more clinical trials.

### 4.2 Problems in gene therapy

**Figure 2:**
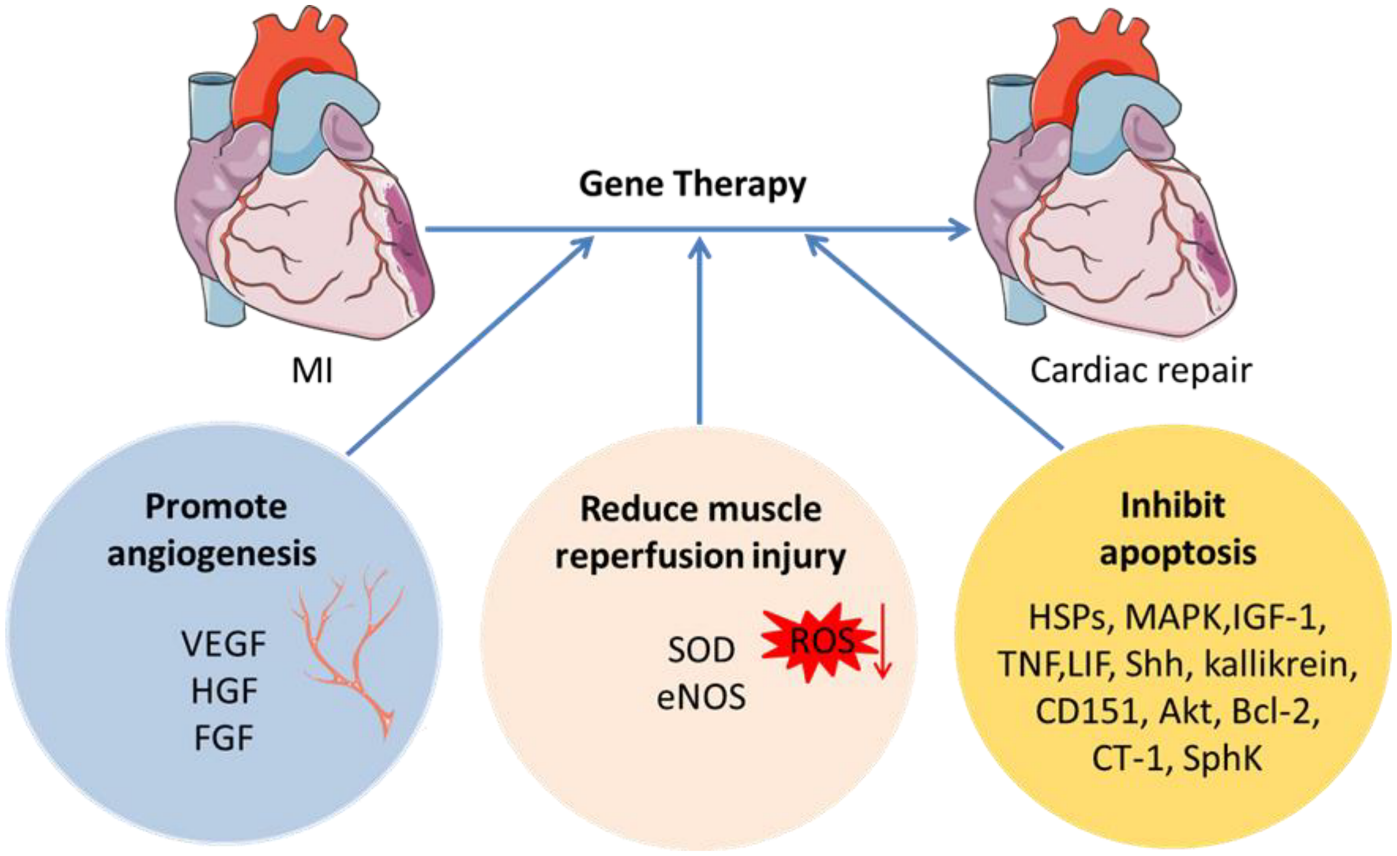
Schematic of gene therapy in MI. Gene therapy improves cardiac function by promoting angiogenesis, reducing muscle reperfusion injury, and inhibiting apoptosis. VEGF, vascular endothelial growth factor; HGF, hepatocyte growth factor; FGF, fibroblast growth factor; ROS, reactive oxygen species; SOD, superoxide dismutase; eNOS, endothelial nitric oxide synthetase; HSPs, heat shock proteins; MAPK, mitogen-activated protein kinase; IGF-1, insulin-like growth factor-1; TNF, tumor necrosis factor; LIF, leukemia inhibitory factor; Shh, sonic hedgehog; CT-1, Cardiac Troponin-1; SphK, sphingosine kinase.

Gene therapy improves cardiac function by promoting angiogenesis, reducing muscle reperfusion injury, and inhibiting apoptosis (Figure 2). However, for most experimental treatments, the safety of gene therapy for ischemic heart disease is very important. Although clinical trials have proved its short-term safety, the long-term observation effect of more than ten years is still lacking. Many confounding factors make it difficult to standardize the grouping of patients in clinical trials, including drug use and the medical environment. When evaluating results, objective rather than subjective evaluation may minimize the impact. The drug-related factors, including dosage, gene transfer efficiency, pharmacokinetics, and pharmacodynamics of individual therapy, must be accurate, because these factors may be different in different patients. The effectiveness analysis of treatment cost should also be considered, because the acquisition of gene therapy vectors is very cumbersome, requiring professional equipment and personnel, and the operation of gene therapy is also invasive to nature. Besides, the cost-benefit ratio of specific gene therapy is not comparable with the existing drugs for ischemic heart disease. Widespread use of small animals for preclinical research may lead to premature overzeal. Gene therapy experiments in large animals will provide a better research perspective. And an orchestration of gene therapy, cell therapy, and biocompatible materials might be a promising research field.

### 4.3 Challenges and limitations

Although stem cell and gene therapy hold immense promise for treating cardiovascular diseases, translating these innovative strategies from the research laboratory to clinical practice presents significant challenges (154). Firstly, the immune system can recognize and attack therapeutic cells or vectors, leading to adverse reactions. Strategies to mitigate immune responses include using immune-privileged sites for cell delivery, modifying vectors to evade immune detection, and transiently suppressing the patient's immune system (155). Additionally, developing personalized therapies using autologous cells can reduce the risk of immune rejection. Precise targeting of therapeutic genes or cells to the affected tissue is crucial but challenging. Off-target effects can lead to unintended consequences. Advances in gene editing technologies, such as CRISPR/Cas9, allow for more precise targeting (156). Combining these technologies with advanced delivery systems, such as viral vectors or nanoparticles, can enhance the accuracy and efficiency of therapeutic delivery. Moreover, navigating the complex regulatory landscape and addressing ethical concerns are critical for clinical translation. Establishing clear guidelines and standards for stem cell and gene therapy research and clinical applications can facilitate regulatory approval (157). Engaging with regulatory bodies early in the development process and ensuring transparency in clinical trials are essential steps (158). As we know, high costs associated with the development and production of stem cell and gene therapies limit their accessibility. Developing cost-effective manufacturing processes and exploring alternative funding models, such as public-private partnerships, can make these therapies more affordable. Additionally, advocating for policies that support the development and reimbursement of novel therapies can improve patient access (159).

Recent years have seen significant progress in the clinical application of stem cell and gene therapy. For example, successful gene therapy trials for inherited blood disorders and certain genetic diseases have demonstrated the potential for long-term clinical benefits. Similarly, advancements in mesenchymal stem cell therapy have shown promise in treating various inflammatory and degenerative conditions (160).

### 4.4 Future directions

#### 4.4.1 Optimizing cell delivery and engraftment

One of the major challenges in stem cell therapy for MI is the poor survival and engraftment of transplanted cells. Future research should focus on optimizing cell delivery methods to enhance cell retention and integration into the damaged myocardium. Techniques such as intramyocardial injection, trans-endocardial delivery, and intracoronary infusion have shown varying degrees of success, but further refinement is needed. Preconditioning stem cells to withstand the ischemic environment, genetic modification to improve cell viability, and the use of paracrine factors to support cell function are being explored. For instance, mesenchymal stem cells (MSCs) can be engineered to secrete higher levels of growth factors and anti-inflammatory cytokines, which can improve their therapeutic efficacy (4, 161). Additionally, combining stem cells with biomaterials or scaffolds can improve cell survival and promote tissue regeneration (2, 162).

#### 4.4.2 Personalized and targeted therapies

The development of personalized therapies tailored to individual patients' needs is a key future direction. Advances in induced pluripotent stem cells (iPSCs) offer the potential for patient-specific cell therapies, reducing the risk of immune rejection (163). Additionally, gene editing technologies such as CRISPR/Cas9 can be used to correct genetic defects or enhance the therapeutic properties of cells (164). Targeted delivery systems, including nanoparticles and exosomes, can improve the precision of gene therapy, ensuring that therapeutic genes are expressed specifically in the damaged myocardium.

#### 4.4.3 Large animal models and clinical trials

Preclinical studies in large animal models are essential for validating the efficacy and safety of stem cell and gene therapies before clinical translation. Large animals such as pigs and non-human primates share similar pathophysiology with humans, making them valuable for testing delivery strategies and assessing long-term outcomes (7, 165). Well-designed clinical trials, including randomized controlled trials with standardized protocols, are also crucial for evaluating the therapeutic potential of these interventions in humans.

## 5 Conclusion

Stem cell and gene therapy represent transformative approaches in the treatment of myocardial infarction, offering hope for improved cardiac function and reduced morbidity. While significant progress has been made, continued research and development are necessary to refine these therapies and address the remaining challenges. By leveraging advancements in technology, optimizing clinical protocols, and fostering collaborative efforts, we can pave the way for these innovative treatments to become standard clinical practices. The future of MI treatment lies in the successful translation of stem cell and gene therapy from the laboratory to the bedside, ultimately improving patient outcomes and quality of life.
